# [1,1′-Bis(diphenyl­phosphino)ferrocene]carbon­yl[dihydro­bis(pyrazol-1-yl)borato]hydridoruthenium(II) acetone solvate

**DOI:** 10.1107/S1600536808037100

**Published:** 2008-11-13

**Authors:** Seong Huh, Alan J. Lough

**Affiliations:** a Department of Chemistry, Hankuk University of Foreign Studies, Yongin 449-791, Republic of Korea; b Department of Chemistry, University of Toronto, 80 St George Street, Toronto, Ontario, Canada M5S 3H6

## Abstract

In the title compound, [FeRu(C_17_H_14_P)_2_(C_6_H_8_BN_4_)H(CO)]·C_3_H_6_O, the Ru^II^ ion is coordinated in a distorted octa­hedral environment involving a hydride ligand, a carbonyl ligand and two bidentate ligands. Of the two bidentate ligands, the bulky 1,1′-bis­(diphenyl­phosphino)ferrocene (dppf) ligand chelates with a larger bite angle of 101.90 (2)°, whereas the bite angle of the [H_2_Bpz_2_]^−^ ligand (pz = pyrazol­yl) is 85.67 (7)°. The latter ligand creates an RuN_4_B six-membered ring with a boat conformation, which puckers towards the site of the small hydride ligand. The hydride ligand is *cis* with respect to the carbonyl ligand and *trans* to one of the P atoms of the dppf ligand. In the crystal structure, there are weak inter­molecular C—H⋯O hydrogen bonds between complex mol­ecules and acetone solvent mol­ecules.

## Related literature

For background information on Ru^II^ complexes, see: Buriez *et al.* (1999 ▶); Han *et al.* (1996 ▶); Hill *et al.* (1998 ▶); Huh *et al.* (1996 ▶); Na *et al.* (1996 ▶); Sánchez-Delgado *et al.* (1986 ▶). For related structures, see: Huh *et al.* (1999 ▶, 2000 ▶).
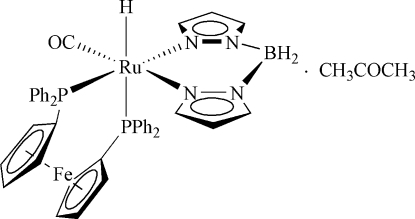


## Experimental

### 

#### Crystal data

[FeRu(C_17_H_14_P)_2_(C_6_H_8_BN_4_)H(CO)]·C_3_H_6_O
*M* *_r_* = 889.49
Monoclinic, 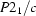

*a* = 9.0730 (2) Å
*b* = 29.9785 (5) Å
*c* = 14.7960 (3) Å
β = 94.617 (1)°
*V* = 4011.38 (14) Å^3^
*Z* = 4
Mo *K*α radiation
μ = 0.86 mm^−1^
*T* = 100 (1) K
0.20 × 0.20 × 0.12 mm

#### Data collection

Nonius KappaCCD diffractometer
Absorption correction: multi-scan (**DENZO-SMN**; Otwinowski & Minor, 1997 ▶) *T*_min_ = 0.847, *T*_max_ = 0.904
41892 measured reflections
11681 independent reflections
7603 reflections with *I* > 2σ(*I*)
*R*_int_ = 0.064

#### Refinement

*R*[*F* ^2^ > 2σ(*F* ^2^)] = 0.038
*wR*(*F* ^2^) = 0.079
*S* = 0.94
11681 reflections
508 parameters
H atoms treated by a mixture of independent and constrained refinement
Δρ_max_ = 0.60 e Å^−3^
Δρ_min_ = −0.50 e Å^−3^

### 

Data collection: *COLLECT* (Nonius, 2002 ▶); cell refinement: *DENZO-SMN* (Otwinowski & Minor, 1997 ▶); data reduction: *DENZO-SMN*; program(s) used to solve structure: *SIR92* (Altomare *et al.*, 1994 ▶); program(s) used to refine structure: *SHELXTL* (Sheldrick, 2008 ▶); molecular graphics: *PLATON* (Spek, 2003 ▶); software used to prepare material for publication: *SHELXTL*.

## Supplementary Material

Crystal structure: contains datablocks I, global. DOI: 10.1107/S1600536808037100/is2360sup1.cif

Structure factors: contains datablocks I. DOI: 10.1107/S1600536808037100/is2360Isup2.hkl

Additional supplementary materials:  crystallographic information; 3D view; checkCIF report

## Figures and Tables

**Table 1:** Selected bond lengths (Å)

Ru1—H1	1.60 (2)
Ru1—C1	1.832 (2)
Ru1—N1	2.1469 (17)
Ru1—N3	2.1514 (18)
Ru1—P1	2.3025 (6)
Ru1—P2	2.4813 (6)

**Table 2:** Hydrogen-bond geometry (Å, °)

*D*—H⋯*A*	*D*—H	H⋯*A*	*D*⋯*A*	*D*—H⋯*A*
C10—H10*A*⋯O1*S*	1.00	2.45	3.426 (3)	166
C32—H32*A*⋯O1*S*^i^	0.95	2.45	3.251 (3)	142

Symmetry code: (i) 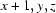.

**Fig. 1.**
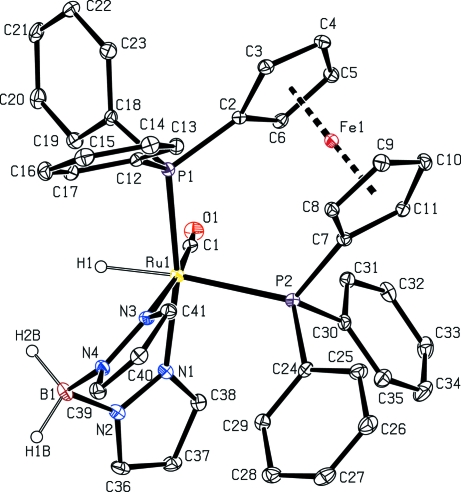
The molecular structure of the title compound with 30% ellipsoids for non-hydrogen atoms. The solvent acetone molecule is not shown and H atoms bonded to C atoms have been omitted.

## supplementary crystallographic information

### Comment

Hydridocarbonyl Ru^II^ polyphophine complexes are excellent catalyst precursors for the homogeneous hydrogenation of carbonyl groups such as aldehydes and ketones under molecular hydrogen atomosphere (Sánchez-Delgado *et al.*, 1986; Huh *et al.*, 1996; Na *et al.*, 1996). Poly(azolyl)borate Ru^II^ complexes are also versatile organometallic catalyst precursors for various organic reactions (Buriez *et al.*, 1999). Most of the poly(azolyl)borate complexes possess potentially tridentate hydrotris(pyrazol-1-yl)borate derivatives (Buriez *et al.*, 1999). However, Hill *et al.* (1998) reported Ru^II^ complexes with a bidentate dihydrobis(pyrazol-1-yl)borate ligand, [H_2_Bpz_2_]^-^ (pz = pyrazolyl), and their reactivity with various unsaturated organic groups. We have also structurally characterized two hydridocarbonyl Ru^II^ complexes bearing a bidentate dihydrobis(pyrazol-1-yl)borate ligand (Huh *et al.*, 1999; 2000). These compounds are potentially efficient catalyst precursors for the homogeneous hydrogenation of aldehydes and ketones.

As the dihydrobis(pyrazol-1-yl)borate ligand usually occupies no more than two coordination sites of a transition metal ion, it may be advantageous to introduce additional chelate ligands to further control the electronic and steric factors of the Ru^II^ complex (Huh *et al.*, 1996). In this regard, we successfully prepared and characterized a new Ru^II^ compound with two different bidentate ligands. A ligand displacement reaction of RuH(PPh_3_)_2_(η^2^-H_2_Bpz_2_)(CO) by 1,1'-bis(diphenylphosphino)ferrocene (dppf) yielded the title compound RuH(CO)(dppf)(η^2^-H_2_Bpz_2_).CH_3_COCH_3_, (I), which contains two different bidentate ligands, a neutral dppf ligand with two P donor atoms and an anionic [H_2_Bpz_2_]^-^ ligand with two N donor atoms. The two mutually *trans*-positioned PPh_3_ ligands of the starting Ru^II^ complex were successfully replaced by the dppf ligand.

The dppf ligand coordinates to the Ru^II^ ion in the normal bidentate chelation mode as shown in Figure 1. The dppf ligand is bulkier than the [H_2_Bpz_2_]^-^ ligand and hence the bite angle for the dppf ligand, P1—Ru1—P2, is 101.90 (2)°. This value is not significantly different from that in the known structure of [RuH(dppf)(NCCH_3_)(PPh_3_)(CO)]^+^, 102.15 (9)° (Han *et al.*, 1996). The N1—Ru1—N3 bite angle is much smaller with a value of 85.67 (7)° A six-membered ring consisting of atoms of Ru1/N1—N4/B1 is in a boat conformation which is puckered towards the hydride ligand.

The longer Ru1—P2 bond (compared to Ru1—P1) may be attributed to the stronger *trans* effect of the hydride ligand than nitrogen donor atom of [H_2_Bpz_2_]^-^. The overall coordination environment of Ru^II^ ion is a distorted octahedral geometry. The two cyclopentadienyl rings of the dppf ligand show a staggered geometry.

### Experimental

A reaction mixture of RuH(PPh_3_)_2_(η^2^-H_2_Bpz_2_)(CO) (207 mg, 0.258 mmol; Hill *et al.* 1998) and dppf (213 mg, 0.516 mmol) in 40 ml CH_2_Cl_2_ was heated under reflux in a nitrogen atmosphere for 22 h. The resulting solution was evaporated to dryness after filtration. The crude solids were dissolved in a minimum amount of acetone and stored at 258 K. X-ray quality yellow block crystals were obtained. The crystals were collected, washed with hexane and air-dried. Anal. Calcd for C_41_H_37_BN_4_FeOP_2_Ru.C_3_H_6_O: C, 59.41; H, 4.87; N, 6.30. Found: C, 59.32; H, 4.77; N, 6.23. IR (KBr): ν(BH_2_) 2408, 2365, 2342, 2279, ν(CO) 1924 cm^-1. 1^H NMR (CDCl_3_, 298 K, TMS, Bruker AMX 500, 500 MHz): δ -6.28 (dd, 1H, ^2^J = 127.9, 26.4 Hz) p.p.m..

### Refinement

H atoms bonded to C atoms were placed in calculated positions, with C—H = 0.95 – 1.00 Å and were included in a riding-model approximation with *U*_iso_(H) = 1.2*U*_eq_(C) or 1.5*U*_eq_(C) for methyl H atoms. The positional parameters of the H atoms bonded to B1 were refined with *U*_iso_(H) = 1.2*U*_eq_(B), while the hydride H atom (H1) was refined independently with an isotropic displacement parameter.

### Crystal data

**Table:**

[FeRu(C_17_H_14_P_1_)_2_(C_6_H_8_BN_4_)H(CO)]·C_3_H_6_O	*F*_000_ = 1824
*M**_r_* = 889.49	*D*_x_ = 1.473 Mg m^−^^3^
Monoclinic, *P*2_1_/*c*	Mo *K*α radiation λ = 0.71073 Å
Hall symbol: -P 2ybc	Cell parameters from 41892 reflections
*a* = 9.0730 (2) Å	θ = 2.6–30.1º
*b* = 29.9785 (5) Å	µ = 0.86 mm^−^^1^
*c* = 14.7960 (3) Å	*T* = 100 (1) K
β = 94.617 (1)º	Plate, yellow
*V* = 4011.38 (14) Å^3^	0.20 × 0.20 × 0.12 mm
*Z* = 4	

### Data collection

**Table:**

Nonius KappaCCD diffractometer	11681 independent reflections
Radiation source: fine-focus sealed tube	7603 reflections with *I* > 2σ(*I*)
Monochromator: graphite	*R*_int_ = 0.064
Detector resolution: 9 pixels mm^-1^	θ_max_ = 30.1º
*T* = 100(1) K	θ_min_ = 2.6º
φ scans and ω scans with κ offsets	*h* = −12→12
Absorption correction: multi-scan(*DENZO-SMN*; Otwinowski & Minor, 1997)	*k* = −42→42
*T*_min_ = 0.847, *T*_max_ = 0.904	*l* = −20→20
41892 measured reflections	

### Refinement

**Table:**

Refinement on *F*^2^	Secondary atom site location: difference Fourier map
Least-squares matrix: full	Hydrogen site location: inferred from neighbouring sites
*R*[*F*^2^ > 2σ(*F*^2^)] = 0.038	H atoms treated by a mixture of independent and constrained refinement
*wR*(*F*^2^) = 0.079	*w* = 1/[σ^2^(*F*_o_^2^) + (0.0318*P*)^2^] where *P* = (*F*_o_^2^ + 2*F*_c_^2^)/3
*S* = 0.94	(Δ/σ)_max_ = 0.001
11681 reflections	Δρ_max_ = 0.60 e Å^−^^3^
508 parameters	Δρ_min_ = −0.50 e Å^−^^3^
Primary atom site location: structure-invariant direct methods	Extinction correction: none

### Special details

**Table:**

Geometry. All e.s.d.'s (except the e.s.d. in the dihedral angle between two l.s. planes) are estimated using the full covariance matrix. The cell e.s.d.'s are taken into account individually in the estimation of e.s.d.'s in distances, angles and torsion angles; correlations between e.s.d.'s in cell parameters are only used when they are defined by crystal symmetry. An approximate (isotropic) treatment of cell e.s.d.'s is used for estimating e.s.d.'s involving l.s. planes.
Refinement. Refinement of *F*^2^ against ALL reflections. The weighted *R*-factor *wR* and goodness of fit *S* are based on *F*^2^, conventional *R*-factors *R* are based on *F*, with *F* set to zero for negative *F*^2^. The threshold expression of *F*^2^ > σ(*F*^2^) is used only for calculating *R*-factors(gt) *etc*. and is not relevant to the choice of reflections for refinement. *R*-factors based on *F*^2^ are statistically about twice as large as those based on *F*, and *R*- factors based on ALL data will be even larger.

### Fractional atomic coordinates and isotropic or equivalent isotropic displacement parameters (Å^2^)

**Table:**

	*x*	*y*	*z*	*U*_iso_*/*U*_eq_	
Ru1	0.595588 (19)	0.361280 (6)	0.185146 (12)	0.01354 (5)	
H1	0.647 (2)	0.3263 (6)	0.1126 (13)	0.019 (6)*	
Fe1	0.27014 (3)	0.468086 (10)	0.17021 (2)	0.01479 (8)	
P1	0.41814 (6)	0.377120 (18)	0.06951 (4)	0.01403 (12)	
P2	0.51433 (7)	0.408011 (18)	0.31033 (4)	0.01519 (13)	
O1	0.80746 (18)	0.42485 (5)	0.10937 (10)	0.0230 (4)	
N1	0.78142 (19)	0.33846 (6)	0.27188 (12)	0.0157 (4)	
N2	0.8112 (2)	0.29422 (6)	0.28533 (12)	0.0178 (4)	
N3	0.4803 (2)	0.30479 (6)	0.23495 (12)	0.0156 (4)	
N4	0.5526 (2)	0.26580 (6)	0.25653 (12)	0.0168 (4)	
C1	0.7193 (2)	0.40206 (7)	0.13852 (15)	0.0162 (5)	
C2	0.3400 (2)	0.43298 (7)	0.06501 (14)	0.0149 (5)	
C3	0.1942 (3)	0.45016 (7)	0.04071 (15)	0.0189 (5)	
H3A	0.1040	0.4319	0.0236	0.023*	
C4	0.1991 (3)	0.49724 (7)	0.04804 (15)	0.0206 (5)	
H4A	0.1127	0.5178	0.0375	0.025*	
C5	0.3462 (3)	0.51024 (7)	0.07443 (15)	0.0184 (5)	
H5A	0.3818	0.5415	0.0852	0.022*	
C6	0.4337 (2)	0.47123 (7)	0.08458 (14)	0.0160 (5)	
H6A	0.5422	0.4703	0.1028	0.019*	
C7	0.3510 (2)	0.44297 (7)	0.29240 (14)	0.0159 (5)	
C8	0.2046 (2)	0.42628 (7)	0.26797 (14)	0.0166 (5)	
H8A	0.1770	0.3942	0.2592	0.020*	
C9	0.1059 (3)	0.46316 (7)	0.25763 (15)	0.0198 (5)	
H9A	−0.0026	0.4614	0.2398	0.024*	
C10	0.1886 (3)	0.50291 (7)	0.27475 (15)	0.0193 (5)	
H10A	0.1483	0.5340	0.2713	0.023*	
C11	0.3385 (3)	0.49088 (7)	0.29740 (14)	0.0180 (5)	
H11A	0.4219	0.5121	0.3124	0.022*	
C12	0.2620 (2)	0.33857 (7)	0.05801 (14)	0.0159 (5)	
C13	0.1236 (2)	0.34714 (7)	0.08908 (15)	0.0184 (5)	
H13A	0.1039	0.3757	0.1133	0.022*	
C14	0.0141 (3)	0.31475 (7)	0.08521 (15)	0.0209 (5)	
H14A	−0.0796	0.3212	0.1064	0.025*	
C15	0.0417 (3)	0.27301 (8)	0.05045 (16)	0.0236 (6)	
H15A	−0.0333	0.2508	0.0473	0.028*	
C16	0.1784 (3)	0.26361 (7)	0.02030 (16)	0.0224 (5)	
H16A	0.1974	0.2348	−0.0030	0.027*	
C17	0.2882 (3)	0.29595 (7)	0.02387 (15)	0.0194 (5)	
H17A	0.3819	0.2891	0.0030	0.023*	
C18	0.4825 (2)	0.37586 (7)	−0.04626 (14)	0.0153 (5)	
C19	0.6268 (3)	0.36541 (7)	−0.06363 (15)	0.0194 (5)	
H19A	0.6941	0.3552	−0.0156	0.023*	
C20	0.6739 (3)	0.36966 (7)	−0.15022 (16)	0.0235 (6)	
H20A	0.7731	0.3626	−0.1609	0.028*	
C21	0.5763 (3)	0.38415 (7)	−0.22115 (16)	0.0253 (6)	
H21A	0.6087	0.3873	−0.2803	0.030*	
C22	0.4321 (3)	0.39395 (8)	−0.20531 (15)	0.0247 (6)	
H22A	0.3647	0.4034	−0.2540	0.030*	
C23	0.3849 (3)	0.39010 (7)	−0.11906 (15)	0.0215 (5)	
H23A	0.2854	0.3972	−0.1090	0.026*	
C24	0.4732 (2)	0.37769 (7)	0.41384 (14)	0.0168 (5)	
C25	0.3670 (3)	0.39273 (8)	0.46939 (15)	0.0220 (5)	
H25A	0.3122	0.4190	0.4534	0.026*	
C26	0.3400 (3)	0.36973 (8)	0.54809 (16)	0.0272 (6)	
H26A	0.2678	0.3806	0.5856	0.033*	
C27	0.4174 (3)	0.33129 (8)	0.57210 (16)	0.0263 (6)	
H27A	0.3982	0.3155	0.6256	0.032*	
C28	0.5235 (3)	0.31590 (8)	0.51712 (16)	0.0242 (6)	
H28A	0.5772	0.2895	0.5330	0.029*	
C29	0.5515 (3)	0.33896 (7)	0.43905 (15)	0.0197 (5)	
H29A	0.6250	0.3283	0.4022	0.024*	
C30	0.6473 (2)	0.45032 (7)	0.35541 (15)	0.0169 (5)	
C31	0.7036 (2)	0.48054 (7)	0.29513 (16)	0.0190 (5)	
H31A	0.6760	0.4777	0.2321	0.023*	
C32	0.7987 (3)	0.51464 (7)	0.32510 (17)	0.0220 (5)	
H32A	0.8343	0.5352	0.2831	0.026*	
C33	0.8414 (3)	0.51844 (8)	0.41672 (17)	0.0267 (6)	
H33A	0.9073	0.5415	0.4377	0.032*	
C34	0.7880 (3)	0.48861 (8)	0.47795 (17)	0.0300 (6)	
H34A	0.8178	0.4912	0.5408	0.036*	
C35	0.6910 (3)	0.45492 (8)	0.44738 (16)	0.0239 (5)	
H35A	0.6540	0.4348	0.4897	0.029*	
C36	0.9347 (3)	0.28986 (8)	0.34064 (15)	0.0210 (5)	
H36A	0.9779	0.2624	0.3608	0.025*	
C37	0.9894 (2)	0.33193 (8)	0.36373 (15)	0.0220 (5)	
H37A	1.0757	0.3391	0.4016	0.026*	
C38	0.8894 (2)	0.36109 (7)	0.31905 (15)	0.0182 (5)	
H38A	0.8966	0.3927	0.3215	0.022*	
C39	0.4607 (3)	0.23805 (7)	0.29611 (15)	0.0203 (5)	
H39A	0.4850	0.2089	0.3175	0.024*	
C40	0.3255 (3)	0.25860 (7)	0.30071 (15)	0.0198 (5)	
H40A	0.2396	0.2470	0.3249	0.024*	
C41	0.3438 (2)	0.30025 (7)	0.26178 (14)	0.0175 (5)	
H41A	0.2692	0.3225	0.2551	0.021*	
B1	0.7149 (3)	0.25689 (9)	0.2369 (2)	0.0198 (6)	
H1B	0.751 (2)	0.2240 (7)	0.2692 (15)	0.024*	
H2B	0.729 (2)	0.2576 (7)	0.1595 (15)	0.024*	
O1S	−0.0065 (2)	0.60075 (6)	0.27422 (15)	0.0531 (6)	
C1S	0.0106 (3)	0.63712 (8)	0.30926 (19)	0.0302 (6)	
C2S	0.1145 (4)	0.64454 (11)	0.3906 (2)	0.0562 (9)	
H2SA	0.1604	0.6161	0.4099	0.084*	
H2SB	0.0602	0.6566	0.4398	0.084*	
H2SC	0.1913	0.6657	0.3759	0.084*	
C3S	−0.0704 (3)	0.67699 (8)	0.2707 (2)	0.0395 (7)	
H3SA	−0.1237	0.6691	0.2127	0.059*	
H3SB	0.0004	0.7009	0.2611	0.059*	
H3SC	−0.1410	0.6872	0.3130	0.059*	

### Atomic displacement parameters (Å^2^)

**Table:**

	*U*^11^	*U*^22^	*U*^33^	*U*^12^	*U*^13^	*U*^23^
Ru1	0.01350 (9)	0.01260 (9)	0.01445 (9)	0.00019 (7)	0.00074 (7)	0.00110 (8)
Fe1	0.01595 (17)	0.01349 (16)	0.01497 (17)	0.00123 (13)	0.00153 (13)	0.00056 (13)
P1	0.0147 (3)	0.0129 (3)	0.0144 (3)	0.0005 (2)	0.0006 (2)	−0.0003 (2)
P2	0.0161 (3)	0.0150 (3)	0.0145 (3)	0.0005 (2)	0.0008 (2)	0.0004 (2)
O1	0.0221 (9)	0.0219 (9)	0.0253 (9)	−0.0046 (7)	0.0030 (7)	0.0032 (7)
N1	0.0143 (10)	0.0148 (9)	0.0181 (10)	0.0003 (8)	0.0024 (8)	0.0014 (8)
N2	0.0166 (10)	0.0162 (10)	0.0208 (10)	0.0021 (8)	0.0023 (8)	0.0034 (8)
N3	0.0165 (10)	0.0153 (9)	0.0148 (10)	−0.0001 (8)	−0.0001 (8)	0.0017 (8)
N4	0.0196 (10)	0.0123 (9)	0.0185 (10)	0.0019 (8)	0.0021 (8)	0.0010 (8)
C1	0.0171 (12)	0.0170 (12)	0.0142 (11)	0.0020 (9)	−0.0015 (9)	−0.0004 (9)
C2	0.0164 (12)	0.0149 (11)	0.0133 (11)	0.0010 (9)	−0.0002 (9)	0.0009 (9)
C3	0.0202 (13)	0.0182 (12)	0.0176 (12)	0.0004 (10)	−0.0030 (10)	−0.0005 (10)
C4	0.0262 (14)	0.0196 (12)	0.0161 (12)	0.0071 (10)	0.0011 (10)	0.0039 (10)
C5	0.0242 (13)	0.0136 (11)	0.0175 (12)	−0.0006 (9)	0.0024 (10)	0.0016 (9)
C6	0.0179 (12)	0.0187 (12)	0.0119 (11)	−0.0023 (9)	0.0038 (9)	0.0009 (9)
C7	0.0181 (12)	0.0156 (11)	0.0144 (12)	0.0018 (9)	0.0030 (9)	0.0024 (9)
C8	0.0197 (12)	0.0143 (11)	0.0162 (12)	0.0011 (9)	0.0031 (9)	0.0008 (9)
C9	0.0156 (12)	0.0237 (13)	0.0207 (13)	0.0020 (10)	0.0059 (10)	0.0015 (10)
C10	0.0194 (13)	0.0184 (12)	0.0206 (13)	0.0049 (10)	0.0051 (10)	−0.0003 (10)
C11	0.0231 (13)	0.0159 (12)	0.0149 (12)	0.0000 (10)	0.0012 (10)	−0.0014 (9)
C12	0.0171 (12)	0.0175 (12)	0.0127 (11)	−0.0003 (9)	−0.0010 (9)	−0.0008 (9)
C13	0.0195 (12)	0.0175 (11)	0.0182 (12)	0.0002 (9)	0.0021 (10)	−0.0011 (10)
C14	0.0171 (12)	0.0243 (13)	0.0216 (13)	−0.0034 (10)	0.0031 (10)	0.0016 (10)
C15	0.0238 (14)	0.0228 (13)	0.0236 (14)	−0.0079 (10)	−0.0016 (11)	0.0015 (11)
C16	0.0282 (14)	0.0153 (12)	0.0236 (13)	−0.0019 (10)	0.0020 (11)	−0.0028 (10)
C17	0.0213 (13)	0.0206 (12)	0.0165 (12)	0.0030 (10)	0.0026 (10)	−0.0004 (10)
C18	0.0220 (13)	0.0103 (10)	0.0140 (11)	−0.0028 (9)	0.0040 (9)	−0.0008 (9)
C19	0.0233 (12)	0.0147 (11)	0.0200 (12)	−0.0028 (10)	0.0010 (10)	0.0015 (10)
C20	0.0273 (14)	0.0205 (13)	0.0239 (13)	−0.0027 (10)	0.0088 (11)	−0.0031 (10)
C21	0.0397 (16)	0.0224 (13)	0.0151 (12)	−0.0035 (11)	0.0101 (11)	−0.0035 (10)
C22	0.0360 (16)	0.0234 (13)	0.0146 (12)	0.0030 (11)	0.0001 (11)	−0.0002 (10)
C23	0.0197 (13)	0.0231 (13)	0.0215 (13)	0.0014 (10)	0.0006 (10)	−0.0020 (10)
C24	0.0196 (12)	0.0181 (11)	0.0126 (11)	−0.0022 (9)	0.0009 (9)	−0.0022 (9)
C25	0.0237 (13)	0.0252 (13)	0.0173 (12)	0.0009 (10)	0.0022 (10)	0.0025 (10)
C26	0.0280 (14)	0.0359 (15)	0.0184 (13)	0.0005 (12)	0.0067 (11)	−0.0008 (11)
C27	0.0306 (15)	0.0320 (14)	0.0165 (12)	−0.0096 (12)	0.0023 (11)	0.0043 (11)
C28	0.0309 (15)	0.0191 (12)	0.0218 (13)	−0.0041 (11)	−0.0026 (11)	0.0048 (11)
C29	0.0205 (13)	0.0203 (12)	0.0182 (12)	0.0014 (10)	0.0019 (10)	−0.0016 (10)
C30	0.0146 (12)	0.0176 (12)	0.0185 (12)	0.0031 (9)	0.0006 (9)	−0.0046 (10)
C31	0.0195 (13)	0.0180 (12)	0.0192 (12)	0.0011 (10)	−0.0003 (10)	−0.0022 (10)
C32	0.0183 (12)	0.0157 (12)	0.0323 (14)	0.0006 (10)	0.0047 (11)	−0.0011 (11)
C33	0.0210 (13)	0.0243 (13)	0.0345 (15)	−0.0031 (11)	0.0002 (11)	−0.0122 (12)
C34	0.0287 (15)	0.0396 (15)	0.0208 (13)	−0.0026 (12)	−0.0032 (11)	−0.0094 (12)
C35	0.0258 (14)	0.0264 (13)	0.0196 (13)	0.0001 (11)	0.0024 (11)	−0.0023 (11)
C36	0.0178 (12)	0.0237 (13)	0.0213 (13)	0.0058 (10)	0.0001 (10)	0.0063 (10)
C37	0.0155 (12)	0.0305 (14)	0.0192 (12)	0.0011 (10)	−0.0034 (10)	0.0007 (11)
C38	0.0178 (12)	0.0172 (11)	0.0197 (12)	−0.0010 (10)	0.0027 (9)	−0.0006 (10)
C39	0.0233 (13)	0.0142 (11)	0.0232 (13)	−0.0028 (10)	0.0007 (10)	0.0015 (10)
C40	0.0193 (13)	0.0210 (12)	0.0191 (12)	−0.0044 (10)	0.0026 (10)	0.0004 (10)
C41	0.0166 (12)	0.0177 (12)	0.0179 (12)	−0.0003 (9)	0.0002 (9)	0.0008 (10)
B1	0.0185 (14)	0.0138 (13)	0.0273 (16)	0.0021 (11)	0.0036 (12)	0.0004 (12)
O1S	0.0455 (14)	0.0234 (11)	0.0910 (18)	−0.0002 (9)	0.0086 (12)	−0.0025 (11)
C1S	0.0219 (13)	0.0239 (14)	0.0468 (17)	0.0003 (11)	0.0154 (12)	0.0070 (13)
C2S	0.058 (2)	0.063 (2)	0.046 (2)	0.0098 (18)	−0.0029 (17)	0.0101 (17)
C3S	0.0322 (17)	0.0273 (15)	0.059 (2)	0.0012 (12)	0.0009 (15)	0.0055 (14)

### Geometric parameters (Å, °)

**Table:**

Ru1—H1	1.60 (2)	C16—C17	1.388 (3)
Ru1—C1	1.832 (2)	C16—H16A	0.9500
Ru1—N1	2.1469 (17)	C17—H17A	0.9500
Ru1—N3	2.1514 (18)	C18—C19	1.390 (3)
Ru1—P1	2.3025 (6)	C18—C23	1.405 (3)
Ru1—P2	2.4813 (6)	C19—C20	1.389 (3)
Fe1—C2	2.023 (2)	C19—H19A	0.9500
Fe1—C6	2.030 (2)	C20—C21	1.387 (3)
Fe1—C8	2.038 (2)	C20—H20A	0.9500
Fe1—C7	2.040 (2)	C21—C22	1.380 (3)
Fe1—C11	2.051 (2)	C21—H21A	0.9500
Fe1—C10	2.053 (2)	C22—C23	1.383 (3)
Fe1—C3	2.055 (2)	C22—H22A	0.9500
Fe1—C9	2.055 (2)	C23—H23A	0.9500
Fe1—C5	2.059 (2)	C24—C25	1.391 (3)
Fe1—C4	2.064 (2)	C24—C29	1.396 (3)
P1—C2	1.818 (2)	C25—C26	1.392 (3)
P1—C12	1.825 (2)	C25—H25A	0.9500
P1—C18	1.854 (2)	C26—C27	1.381 (3)
P2—C7	1.817 (2)	C26—H26A	0.9500
P2—C30	1.838 (2)	C27—C28	1.389 (3)
P2—C24	1.845 (2)	C27—H27A	0.9500
O1—C1	1.161 (3)	C28—C29	1.387 (3)
N1—C38	1.341 (3)	C28—H28A	0.9500
N1—N2	1.365 (2)	C29—H29A	0.9500
N2—C36	1.340 (3)	C30—C35	1.393 (3)
N2—B1	1.558 (3)	C30—C31	1.396 (3)
N3—C41	1.338 (3)	C31—C32	1.388 (3)
N3—N4	1.365 (2)	C31—H31A	0.9500
N4—C39	1.345 (3)	C32—C33	1.384 (3)
N4—B1	1.547 (3)	C32—H32A	0.9500
C2—C3	1.438 (3)	C33—C34	1.388 (3)
C2—C6	1.443 (3)	C33—H33A	0.9500
C3—C4	1.416 (3)	C34—C35	1.391 (3)
C3—H3A	1.0000	C34—H34A	0.9500
C4—C5	1.415 (3)	C35—H35A	0.9500
C4—H4A	1.0000	C36—C37	1.388 (3)
C5—C6	1.415 (3)	C36—H36A	0.9500
C5—H5A	1.0000	C37—C38	1.388 (3)
C6—H6A	1.0000	C37—H37A	0.9500
C7—C8	1.438 (3)	C38—H38A	0.9500
C7—C11	1.443 (3)	C39—C40	1.379 (3)
C8—C9	1.423 (3)	C39—H39A	0.9500
C8—H8A	1.0000	C40—C41	1.391 (3)
C9—C10	1.420 (3)	C40—H40A	0.9500
C9—H9A	1.0000	C41—H41A	0.9500
C10—C11	1.421 (3)	B1—H1B	1.13 (2)
C10—H10A	1.0000	B1—H2B	1.16 (2)
C11—H11A	1.0000	O1S—C1S	1.212 (3)
C12—C13	1.396 (3)	C1S—C2S	1.485 (4)
C12—C17	1.401 (3)	C1S—C3S	1.492 (3)
C13—C14	1.386 (3)	C2S—H2SA	0.9800
C13—H13A	0.9500	C2S—H2SB	0.9800
C14—C15	1.383 (3)	C2S—H2SC	0.9800
C14—H14A	0.9500	C3S—H3SA	0.9800
C15—C16	1.380 (3)	C3S—H3SB	0.9800
C15—H15A	0.9500	C3S—H3SC	0.9800
			
H1—Ru1—C1	87.9 (7)	C10—C9—Fe1	69.69 (13)
H1—Ru1—N1	86.2 (7)	C8—C9—Fe1	69.01 (13)
C1—Ru1—N1	87.83 (8)	C10—C9—H9A	125.8
H1—Ru1—N3	83.7 (7)	C8—C9—H9A	125.8
C1—Ru1—N3	169.69 (8)	Fe1—C9—H9A	125.8
N1—Ru1—N3	85.67 (7)	C9—C10—C11	108.11 (19)
H1—Ru1—P1	82.0 (7)	C9—C10—Fe1	69.88 (13)
C1—Ru1—P1	89.75 (7)	C11—C10—Fe1	69.66 (12)
N1—Ru1—P1	168.07 (5)	C9—C10—H10A	125.9
N3—Ru1—P1	94.98 (5)	C11—C10—H10A	125.9
H1—Ru1—P2	173.2 (7)	Fe1—C10—H10A	125.9
C1—Ru1—P2	97.60 (7)	C10—C11—C7	108.5 (2)
N1—Ru1—P2	90.00 (5)	C10—C11—Fe1	69.82 (13)
N3—Ru1—P2	90.39 (5)	C7—C11—Fe1	68.93 (12)
P1—Ru1—P2	101.90 (2)	C10—C11—H11A	125.7
C2—Fe1—C6	41.71 (8)	C7—C11—H11A	125.7
C2—Fe1—C8	110.71 (9)	Fe1—C11—H11A	125.7
C6—Fe1—C8	137.83 (9)	C13—C12—C17	118.0 (2)
C2—Fe1—C7	112.17 (8)	C13—C12—P1	124.40 (16)
C6—Fe1—C7	109.97 (9)	C17—C12—P1	117.27 (17)
C8—Fe1—C7	41.29 (8)	C14—C13—C12	121.2 (2)
C2—Fe1—C11	142.11 (9)	C14—C13—H13A	119.4
C6—Fe1—C11	112.02 (9)	C12—C13—H13A	119.4
C8—Fe1—C11	68.75 (9)	C15—C14—C13	119.9 (2)
C7—Fe1—C11	41.32 (8)	C15—C14—H14A	120.0
C2—Fe1—C10	177.15 (9)	C13—C14—H14A	120.0
C6—Fe1—C10	140.60 (9)	C16—C15—C14	119.9 (2)
C8—Fe1—C10	68.57 (9)	C16—C15—H15A	120.0
C7—Fe1—C10	69.25 (9)	C14—C15—H15A	120.0
C11—Fe1—C10	40.52 (9)	C15—C16—C17	120.4 (2)
C2—Fe1—C3	41.30 (8)	C15—C16—H16A	119.8
C6—Fe1—C3	68.74 (9)	C17—C16—H16A	119.8
C8—Fe1—C3	113.94 (9)	C16—C17—C12	120.6 (2)
C7—Fe1—C3	143.10 (9)	C16—C17—H17A	119.7
C11—Fe1—C3	175.43 (8)	C12—C17—H17A	119.7
C10—Fe1—C3	136.17 (9)	C19—C18—C23	118.1 (2)
C2—Fe1—C9	137.30 (9)	C19—C18—P1	123.14 (17)
C6—Fe1—C9	178.48 (9)	C23—C18—P1	118.44 (17)
C8—Fe1—C9	40.69 (8)	C20—C19—C18	121.0 (2)
C7—Fe1—C9	69.08 (9)	C20—C19—H19A	119.5
C11—Fe1—C9	68.13 (9)	C18—C19—H19A	119.5
C10—Fe1—C9	40.43 (9)	C21—C20—C19	120.1 (2)
C3—Fe1—C9	111.24 (9)	C21—C20—H20A	119.9
C2—Fe1—C5	69.29 (9)	C19—C20—H20A	119.9
C6—Fe1—C5	40.48 (8)	C22—C21—C20	119.6 (2)
C8—Fe1—C5	177.30 (9)	C22—C21—H21A	120.2
C7—Fe1—C5	136.06 (9)	C20—C21—H21A	120.2
C11—Fe1—C5	109.49 (9)	C21—C22—C23	120.5 (2)
C10—Fe1—C5	111.57 (9)	C21—C22—H22A	119.8
C3—Fe1—C5	67.95 (9)	C23—C22—H22A	119.8
C9—Fe1—C5	141.02 (9)	C22—C23—C18	120.6 (2)
C2—Fe1—C4	68.99 (9)	C22—C23—H23A	119.7
C6—Fe1—C4	68.04 (9)	C18—C23—H23A	119.7
C8—Fe1—C4	142.53 (9)	C25—C24—C29	118.3 (2)
C7—Fe1—C4	175.85 (9)	C25—C24—P2	121.78 (17)
C11—Fe1—C4	135.46 (9)	C29—C24—P2	119.95 (17)
C10—Fe1—C4	109.79 (9)	C24—C25—C26	120.7 (2)
C3—Fe1—C4	40.22 (8)	C24—C25—H25A	119.7
C9—Fe1—C4	112.99 (9)	C26—C25—H25A	119.7
C5—Fe1—C4	40.14 (9)	C27—C26—C25	120.6 (2)
C2—P1—C12	106.41 (10)	C27—C26—H26A	119.7
C2—P1—C18	97.80 (10)	C25—C26—H26A	119.7
C12—P1—C18	101.58 (10)	C26—C27—C28	119.2 (2)
C2—P1—Ru1	117.68 (7)	C26—C27—H27A	120.4
C12—P1—Ru1	115.45 (7)	C28—C27—H27A	120.4
C18—P1—Ru1	115.41 (8)	C29—C28—C27	120.3 (2)
C7—P2—C30	99.13 (10)	C29—C28—H28A	119.9
C7—P2—C24	100.87 (10)	C27—C28—H28A	119.9
C30—P2—C24	102.09 (10)	C28—C29—C24	120.9 (2)
C7—P2—Ru1	120.30 (7)	C28—C29—H29A	119.5
C30—P2—Ru1	115.76 (8)	C24—C29—H29A	119.5
C24—P2—Ru1	115.69 (7)	C35—C30—C31	118.0 (2)
C38—N1—N2	106.74 (17)	C35—C30—P2	123.33 (18)
C38—N1—Ru1	130.98 (15)	C31—C30—P2	118.62 (17)
N2—N1—Ru1	122.24 (13)	C32—C31—C30	121.6 (2)
C36—N2—N1	109.25 (18)	C32—C31—H31A	119.2
C36—N2—B1	128.41 (19)	C30—C31—H31A	119.2
N1—N2—B1	122.27 (18)	C33—C32—C31	119.4 (2)
C41—N3—N4	106.40 (17)	C33—C32—H32A	120.3
C41—N3—Ru1	131.99 (14)	C31—C32—H32A	120.3
N4—N3—Ru1	121.08 (14)	C32—C33—C34	120.1 (2)
C39—N4—N3	109.17 (18)	C32—C33—H33A	119.9
C39—N4—B1	127.43 (19)	C34—C33—H33A	119.9
N3—N4—B1	123.40 (18)	C33—C34—C35	120.0 (2)
O1—C1—Ru1	173.74 (19)	C33—C34—H34A	120.0
C3—C2—C6	106.32 (18)	C35—C34—H34A	120.0
C3—C2—P1	133.40 (17)	C34—C35—C30	120.8 (2)
C6—C2—P1	120.21 (16)	C34—C35—H35A	119.6
C3—C2—Fe1	70.55 (12)	C30—C35—H35A	119.6
C6—C2—Fe1	69.40 (12)	N2—C36—C37	109.1 (2)
P1—C2—Fe1	126.63 (11)	N2—C36—H36A	125.5
C4—C3—C2	108.4 (2)	C37—C36—H36A	125.5
C4—C3—Fe1	70.23 (12)	C36—C37—C38	104.4 (2)
C2—C3—Fe1	68.15 (12)	C36—C37—H37A	127.8
C4—C3—H3A	125.8	C38—C37—H37A	127.8
C2—C3—H3A	125.8	N1—C38—C37	110.6 (2)
Fe1—C3—H3A	125.8	N1—C38—H38A	124.7
C5—C4—C3	108.6 (2)	C37—C38—H38A	124.7
C5—C4—Fe1	69.74 (13)	N4—C39—C40	109.3 (2)
C3—C4—Fe1	69.56 (12)	N4—C39—H39A	125.4
C5—C4—H4A	125.7	C40—C39—H39A	125.4
C3—C4—H4A	125.7	C39—C40—C41	104.2 (2)
Fe1—C4—H4A	125.7	C39—C40—H40A	127.9
C6—C5—C4	108.09 (19)	C41—C40—H40A	127.9
C6—C5—Fe1	68.66 (12)	N3—C41—C40	111.0 (2)
C4—C5—Fe1	70.11 (13)	N3—C41—H41A	124.5
C6—C5—H5A	125.9	C40—C41—H41A	124.5
C4—C5—H5A	125.9	H1B—B1—H2B	112.4 (15)
Fe1—C5—H5A	125.9	H1B—B1—N4	108.6 (11)
C5—C6—C2	108.60 (19)	H2B—B1—N4	111.2 (11)
C5—C6—Fe1	70.87 (13)	H1B—B1—N2	107.4 (11)
C2—C6—Fe1	68.88 (12)	H2B—B1—N2	109.8 (11)
C5—C6—H6A	125.7	N4—B1—N2	107.27 (18)
C2—C6—H6A	125.7	O1S—C1S—C2S	122.2 (3)
Fe1—C6—H6A	125.7	O1S—C1S—C3S	121.0 (3)
C8—C7—C11	106.53 (19)	C2S—C1S—C3S	116.8 (2)
C8—C7—P2	124.19 (16)	C1S—C2S—H2SA	109.5
C11—C7—P2	129.28 (17)	C1S—C2S—H2SB	109.5
C8—C7—Fe1	69.30 (12)	H2SA—C2S—H2SB	109.5
C11—C7—Fe1	69.75 (12)	C1S—C2S—H2SC	109.5
P2—C7—Fe1	124.98 (12)	H2SA—C2S—H2SC	109.5
C9—C8—C7	108.49 (19)	H2SB—C2S—H2SC	109.5
C9—C8—Fe1	70.30 (12)	C1S—C3S—H3SA	109.5
C7—C8—Fe1	69.41 (12)	C1S—C3S—H3SB	109.5
C9—C8—H8A	125.8	H3SA—C3S—H3SB	109.5
C7—C8—H8A	125.8	C1S—C3S—H3SC	109.5
Fe1—C8—H8A	125.8	H3SA—C3S—H3SC	109.5
C10—C9—C8	108.3 (2)	H3SB—C3S—H3SC	109.5

### Hydrogen-bond geometry (Å, °)

**Table:**

*D*—H···*A*	*D*—H	H···*A*	*D*···*A*	*D*—H···*A*
C10—H10A···O1S	1.00	2.45	3.426 (3)	166
C32—H32A···O1S^i^	0.95	2.45	3.251 (3)	142

Symmetry codes: (i) *x*+1, *y*, *z*.

## References

[bb1] Altomare, A., Cascarano, G., Giacovazzo, C., Guagliardi, A., Burla, M. C., Polidori, G. & Camalli, M. (1994). *J. Appl. Cryst.* **27**, 435.

[bb2] Buriez, B., Burns, I. D., Hill, A. F., White, A. J. P., Williams, D. J. & Wilton-Ely, J. D. E. T. (1999). *Organometallics*, **18**, 1504–1516.

[bb3] Han, S.-H., Sung, K.-M., Huh, S., Jun, M.-J., Whang, D. & Kim, K. (1996). *Polyhedron*, **15**, 3811–3820.

[bb4] Hill, A. F., White, A. J. P., Williams, D. J. & Wilton-Ely, J. D. E. T. (1998). *Organometallics*, **17**, 4249–4258.

[bb5] Huh, S., Kim, Y., Park, S., Park, T.-J. & Jun, M.-J. (1999). *Acta Cryst.* C**55**, 850–852.

[bb6] Huh, S., Park, Y. J., Lough, A. J. & Jun, M.-J. (2000). *Acta Cryst.* C**56**, 416–417. 10.1107/s0108270199016649 10815191

[bb7] Huh, S., Sung, K.-M., Cho, Y., Jun, M.-J., Whang, D. & Kim, K. (1996). *Polyhedron*, **15**, 1473–1479.

[bb8] Na, K.-I., Huh, S., Sung, K.-M. & Jun, M.-J. (1996). *Polyhedron*, **15**, 1841–1846.

[bb9] Nonius (2002). *COLLECT* Nonius BV, Delft, The Netherlands.

[bb10] Otwinowski, Z. & Minor, W. (1997). *Methods in Enzymology*, Vol. 276, *Macromolecular Crystallography*, Part A, edited by C. W. Carter Jr & R. M. Sweet, pp. 307–326. New York: Academic Press.

[bb11] Sánchez-Delgado, R. A., Valencia, N., Márquez-Silva, R.-L., Andriollo, A. & Medina, M. (1986). *Inorg. Chem.* **25**, 1106–1111.

[bb12] Sheldrick, G. M. (2008). *Acta Cryst.* A**64**, 112–122. 10.1107/S0108767307043930 18156677

[bb13] Spek, A. L. (2003). *J. Appl. Cryst.* **36**, 7–13.

